# Supplementary material for: Maternal Chronic Ultrasound Stress Provokes Immune Activation and Behavioral Deficits in the Offspring: A Mouse Model of Neurodevelopmental Pathology
Source: Int J Mol Sci. 2023 Jul 20;24(14):11712. doi: 10.3390/ijms241411712 (PMC10380915; doi:10.3390/ijms241411712)
Supplement: Supplementary file 1 [file ijms-24-11712-s001.zip › ijms-2385329-supplementary.pdf]

Supplementary Material

Maternal chronic ultrasound stress exerts immune activation and behavioural deficits in the offspring: a mouse model of neurodevelopmental pathology

Dmitrii Pavlov*, Anna Gorlova, Abrar Haque, Carlos Cavalcante, Evgeniy Svirin, Elizaveta Grigorieva, Elisaveta Sheveleva, Dmitry Malin, Sofia Efimochkina, Andrei Proshin, Aleksei Umriukhin, Sergey Morozov, Tatyana Strekalova

***Correspondence:**

Corresponding Author dmitrii.pavlov1@ucalgary.ca

ULTRASOUND RADIATION

Supplementary Table 1. Ultrasound radiation parameters

Frequency range	Characteristics of stress exposure
20-25 kHz	Low range frequencies with the loudness at 50±5dB. Sporadic appearance within the 10 minutes long timeline; when the time is over a new frequency range is introduced. Low range frequencies were interrupted with above-indicated range frequencies averaged at 70 Hz±10 Hz for 1 second on a random basis. Low range frequencies have semantic meaning in rodent communication listed in supplementary Table 2, while 70 Hz±10 Hz range frequencies do not.
> 25 < 40 kHz	Middle range frequencies with the loudness at 50±5dB. Sporadic appearance within the 10 minutes long timeline, comparable to presence of low range frequencies. Low and middle ranges occupy 70% of total ultrasound exposure time during a day. Accordingly, they were randomly interrupted with above-indicated range frequencies averaged at 70 Hz±10 Hz for 1 second. Middle range frequencies are used in rodent communication, while above-range insertions lack informational value.
40-45 kHz	High range frequencies of the same loudness of 50±5dB. Sporadic appearance within the 10 minutes long timeline, they cover around 30% of the total exposure time on a daily basis. As with low and middle ranges, it was

	sporadically mixed with 1 second long above-indicated range frequencies averaged at 70 Hz±10 Hz. High range frequencies are naturally emitted by rodents to communicate with conspecifics, but not above-range insertions.
> 70 Hz	Sporadically appeared range of frequencies for no longer than 1 second at a time, loudness is set at 50±5dB. They appeared within all three main ultrasonic ranges and served as an additional component of informational unpredictability.

The Weitech device emitted three ranges of frequencies randomly alternating them with each other and the periods of silence. The even distribution of ultrasonic radiation was confirmed with the Discovery Channel ultrasound detector. During the day, low and middle range frequencies constitute 35% of the total emission time; high range frequencies constitute 30% of the total emission time. dB – decibel, Hz – hertz, kHz – kilohertz.

Supplementary Table 2. Ultrasonic communication in rodent studies

Frequency range	Rodent semantic comprehension of context	References
20-25 kHz	Associated with negative emotional state	Kuraoka and Nakamura, 2010
25-45 kHz	Associated with neutral emotional state or produced in some life-threatening conditions, specific ranges and song compositions are context-dependent	Kuraoka and Nakamura, 2010; Takahashi et al., 2010
>50 kHz	Used during physiologically positive experiences, such as mom-pup interaction or mating	Panksepp et al., 2007; Okabe et. Al., 2010

The nature of species-specific information transmitted by mice at the ultrasonic range is a subject of ongoing research worldwide, and the table summarizes well-established semantic contexts where mice emit frequencies within a specific range. Adverse effects of random chronic alternation of listed frequencies have been previously confirmed in studies by Gorlova et al., 2019 and Pavlov et al., 2019 among others. kHz – kilohertz.

QUANTITATIVE REVERSE TRANSCRIPTION POLYMERASE CHAIN REACTION ANALYSIS (QRT-PCR)

Supplementary Table 3. Sequences of primers used for qRT-PCR

Gene	Forward primer 5′–3′	Reverse primer 5′–3′
GAPDH	TGCACCACCAACTGCTTAG	GGATGCAGGGATGATGTTC
IL-1β	CCTCCAGGATGAGGACATGAGCAC	TCATCATCCCATGAGTCACAGAGG
IL-6	TAGTCCTTCCTACCCCAATTTCC	TTGGTCCTTAGCCACTCCTTC
GSK3β	GCACTCTTCAACTTTACCACTCA	CGAGCATGTGGAGGGATAAG

Real-time PCR was performed in the following conditions: initial denaturation step (95 °C, 4 min) followed by 40 cycles of denaturation at 95 °C for 20 seconds, annealing at 54 °C for 90 seconds. Reactions were performed in 10 µl volume using 1 µl of analysed cDNA. All samples were run in duplicates. Sequences of all primers used are listed in Supplementary Table 3.

Supplementary Table 4. Summary of comparisons between mice that underwent ultrasound exposure and control animals in GMCSF, IFNγ, IL-1α, IL-1β, IL-2, IL-3, IL-4, IL-5, IL-6, IL-10, IL-12p70, IL-17, MCP-1, MIP-1α, RANTES, and TNFα protein content. Mann-Whitney test was used (see ms text).

Targets	Groups	
	Control (C)	Ultrasound-exposed (UE)
**GMCSF**	undetected	undetected

**IFNγ**	13.63±3.2	30.71±7.26<br>vs. C: p=0.0952
**IL-1α**	14.24±2.9	26.53±5.95<br>vs. C: p=0.09
**IL-1β**	69.09±2.71	184.45±21.81<br>**vs. C: p=0.079**
**IL-2**	undetected	undetected
**IL-3**	5.86±0.64	7.6±1.56<br>vs. C: p=0.8413
**IL-4**	7.38±1.59	6.7±1.3<br>vs. C: p=0.99
**IL-5**	5.17±1.11	5.85±0.8<br>vs. C: p=0.5476
**IL-6**	9.12±1.87	16.96±2.045<br>**vs. C: p=0.0159**
**IL-10**	9.2±1.8	3.96±0.12<br>**vs. C: p=0.0159**
**IL-12p70**	7.19±1.11	11.93±3<br>vs. C: p=0.1508
**IL-17**	7.41±1.48	13.35±1.26<br>**vs. C: p=0.0317**

**MCP-1**	10.98±2.39	13.72±2.96<br>vs. C: p=0.6905
**MIP-1α**	19.13±1.95	20.47±4.13<br>vs. C: p=0.99
**RANTES**	5.91±1.2	11.29±1.51<br>**vs. C: p=0.0317**
**TNFα**	6.23±1.03	12.14±1.99<br>**vs. C: p=0.0397**

Significant differences are in **bold**. Concentrations are pg/ml. Concentrations of undetected molecules were lower than the detectable threshold.

References

Gorlova A, Pavlov D, Anthony DC, Ponomarev ED, Sambon M, Proshin A, Shafarevich I, Babaevskaya D, Lesch KP, Bettendorff L, Strekalova T. Thiamine and benfotiamine counteract ultrasound-induced aggression, normalize AMPA receptor expression and plasticity markers, and reduce oxidative stress in mice. Neuropharmacology. (2019) 156:107543. doi: 10.1016/j.neuropharm.2019.02.025

Kuraoka K, Nakamura K. Vocalization as a specific trigger of emotional responses. Handbook of Mammalian Vocalization, Elsevier. (2010). 167-177. doi:10.1016/B978-0-12-374593-4.00017-6

Okabe S, Nagasawa M, Kihara T, Kato M, Harada T, Koshida N, Mogi K, Kikusui T. The effects of social experience and gonadal hormones on retrieving behavior of mice and their responses to pup ultrasonic vocalizations. Zoolog Sci. (2010). 27(10):790-5. doi: 10.2108/zsj.27.790

Panksepp JB, Jochman KA, Kim JU, Koy JJ, Wilson ED, Chen Q, Wilson CR, Lahvis GP. Affiliative behavior, ultrasonic communication and social reward are influenced by genetic variation in adolescent mice. PLoS One. (2007). 2(4):e351. doi: 10.1371/journal.pone.0000351

Pavlov D, Bettendorff L, Gorlova A, Olkhovik A, Kalueff AV, Ponomarev ED, Inozemtsev A, Chekhonin V, Lesch KP, Anthony DC, Strekalova T. Neuroinflammation and aberrant hippocampal plasticity in a mouse model of emotional stress evoked by exposure to ultrasound of alternating frequencies. Prog Neuropsychopharmacol Biol Psychiatry. (2019). 90:104-116. doi: 10.1016/j.pnpbp.2018.11.014

Takahashi N, Kashino M, Hironaka N. Structure of rat ultrasonic vocalizations and its relevance to behavior. PLoS One. (2010). 5: 14115. doi: 10.1371/journal.pone.0014115
